# An editor for the generation and customization of geometry restraints

**DOI:** 10.1107/S2059798316016570

**Published:** 2017-02-01

**Authors:** Nigel W. Moriarty, Eli J. Draizen, Paul D. Adams

**Affiliations:** a Molecular Biophysics and Integrated Bioimaging Division, Lawrence Berkeley National Laboratory, One Cyclotron Road, Berkeley, CA 94720, USA; b Department of Bioengineering, University of California Berkeley, Berkeley, CA 94720, USA

**Keywords:** crystallographic refinement, geometric restraints, ideal geometry, graphical user interfaces, *PHENIX*

## Abstract

Chemical restraints for use in macromolecular structure refinement are produced by a variety of methods, including a number of programs that use chemical information to generate the required bond, angle, dihedral, chiral and planar restraints. These programs help to automate the process and therefore minimize the errors that could otherwise occur if it were performed manually. Furthermore, restraint-dictionary generation programs can incorporate chemical and other prior knowledge to provide reasonable choices of types and values. However, the use of restraints to define the geometry of a molecule is an approximation introduced with efficiency in mind. The representation of a bond as a parabolic function is a convenience and does not reflect the true variability in even the simplest of molecules. Another complicating factor is the interplay of the molecule with other parts of the macromolecular model. Finally, difficult situations arise from molecules with rare or unusual moieties that may not have their conformational space fully explored. These factors give rise to the need for an interactive editor for WYSIWYG interactions with the restraints and molecule. *Restraints Editor, Especially Ligands* (*REEL*) is a graphical user interface for simple and error-free editing along with additional features to provide greater control of the restraint dictionaries in macromolecular refinement.

## Abstract 2

Obtaining a restraint dictionary for novel ligands or improving restraints for known ligands can require their manual modification. This can be tedious and error-prone. *REEL* is a restraints editor that provides quick, easy and accurate development, allowing global changes and fine-tuning of individual restraints.

## 1. Introduction   

Restraint dictionaries are used in macromolecular structure refinement to help compensate for the typically low data-to-parameter ratio. Chemical restraints, which form a subclass of all restraints and are the most fundamental, are based on the internal coordinates of a molecular entity and generally include bonds, angles, dihedrals, chirals and planes. Each of the restraints is assigned an ideal value and an estimated standard deviation to define the center and curvature of the parabolic function for calculating the residual and gradient of the restraint (for more details, see Evans, 2007 ▸). A summation of the restraint residuals and the gradients is used as the functional to minimize the geometry term in the structure-refinement process. One limitation of the restraints paradigm is the need for the Cartesian coordinates of the molecule geometry to be consistent with the restraints. A simple example is a poorly modeled chiral center. The gradients from the chiral restraint and the angle restraints involved do not provide a pathway to the global minima of the moiety, but rather minimize to a chemical unreasonable configuration similar to an umbrella that is a local minima in the restraint paradigm but is not so in chemical reality. This is a shortcoming that can surface in poor models that provide chemically inaccurate geometries outside the radius of convergence of the restraints framework.

For the common chemical entities, such as amino acids, nucleotides and common small molecules, the dictionaries have been determined and improved though use and experimentation. The GeoStd restraints library (N. W. Moriarty & P. D. Adams; http://sourceforge.net/projects/geostd) is an open-source project on SourceForge that contains dictionaries that have been modified to accommodate all of the amino-acid rotamer states determined by the Richardson group (Lovell *et al.*, 2000 ▸). Structural changes made to the restraints for rotamers include rotamer descriptions that include the definitions of the torsions, the rotamer name, the torsion values and the frequency. In addition, a field, alt_value_angle, has been introduced to the dihedral loop that lists the acceptable angles of the dihedral to allow each rotamer to be at or near a torsional minimum.

For the less common entities that have been deposited in the Protein Data Bank (Berman *et al.*, 2000 ▸), restraint libraries exist that can be used. For novel ligands and entities that have not been included in restraint libraries, the user has the burden of generating suitable geometry restraints for use in the refinement.

Within the *PHENIX* package (Adams *et al.*, 2010 ▸, 2011 ▸), chemical restraint generation is performed using the *electronic Ligand Builder and Optimization Workbench* (Moriarty *et al.*, 2009 ▸). Known as *eLBOW*, it is designed as a very flexible and powerful tool generating dictionaries that are chemically accurate and consistent with a number of workflows. It is also the workhorse in other programs, for example generating restraints and adding H atoms in the *PHENIX* GUI (Echols *et al.*, 2012 ▸) for *phenix.refine* (Afonine *et al.*, 2012 ▸). Furthermore, *eLBOW* generates dictionaries with additional fields that need to be handled appropriately. These include the formal charge of an atom, a number of informational fields and the nuclear bond distances for neutron refinement.

Ideally, the geometry restraints in the libraries and those generated by the appropriate programs would be sufficient for all cases. Unfortunately, not all of the chemical knowledge about a certain molecule can be accommodated in the chemical format of choice. Some formats do not have three-dimensional Cartesian coordinates [SMILES (Weininger, 1988 ▸) and Mol2D], while others can lack bond-connectivity information (PDB and xyz) that leads to ambiguity about tautomeric states. Conversion of the chemical input information can also lead to data loss given that the restraints model of the molecular geometry is an approximation. For example, an *sp* ^2^-hybridized C atom will remain planar in the vast majority of molecules. The restraints pertaining to the C atom will include three bonds, three angles and, because of limitations of the restraints paradigm, a planar restraint on at least four atoms. Strictly speaking, there is no internal coordinate of a molecule known as ‘planar’, but it is required to enforce planarity in this situation. The reason is that each of the three angles has an ideal value that is constant. If one angle is distorted in a real molecule, the other two will compensate to retain a planar configuration. In the restraints situation, the ideal angle values remain the same, so a distorted angle will not have compensating companion angles and will thus bend out of the plane. A dynamic restraint on *sp* ^2^ centers that restrains the sum of the three angles to be 360° would be an improvement. Dynamic restraints are becoming standard practice in structure refinement: single-value restraints have been shown to be inadequate for the protein backbone, and the Conformation Dependent Library (CDL; Berkholz *et al.*, 2009 ▸) has recently been made the default in *phenix.refine* (Moriarty *et al.*, 2014 ▸, 2016 ▸).

Furthermore, there are moieties that do not have an explicit restraint for controlling their state. In the case of *cis*–*trans* (*E*–*Z*) isomers, some chemical inputs do specify the desired isomer. Without the *E*–*Z* specification, the input geometry, and to a lesser extent the steric hindrance, can guide the refinement. However, in most cases the dictionary will not provide a pathway from the *E*-isomer to the *Z*-isomer, as each is a local minimum. To restrain the molecule to a specific *cis*–*trans* isomer, a torsion restraint with a single minimum (period=1) may be used in the geometry dictionary. Another example is the pucker state of a saturated ring moiety. The pucker state is not specified by any of the common chemical formats, nor is there an implicit restraint. Therefore, the options are to leave flexibility in the dictionaries allowing the density to guide the geometry refinement or to control it indirectly using a set of torsion restraints.

## 2. Concept   

Editors allow the operator to change a computer file. One of the most basic is a text editor that allows the entry and deletion of ASCII characters in a simple text file. The same concept holds for a restraints editor, however here the atomicity of edits is geometry restraints that are being added and deleted. The smallest item that can be added or deleted in a text file is a single character; however, in a restraints editor the smallest item is a single restraint. However, a single restraint can have its internal values changed.

Geometry restraints are based on internal coordinates that are fundamentally different from Cartesian coordinates. To this end, *REEL* is designed to interact with the atoms and internal coordinates and exclude direct control of the Cartesian coordinates. Selection of the desired internal coordinate allows direct modifications of the ideal value and estimated standard deviation. This change needs to be propagated to all restraints appropriately, reflected in the geometry of the molecule and validated for simple errors. There should also be broad controls that can change many of the individual restraints to conform to the requirements of a certain user.

## 3. Details   

### 3.1. Overview of windows and layout   

The Restraints Editor (*REEL*) was developed primarily for editing restraint dictionary files. Hence, the more detailed features of the GUI only relate to the editing of dictionary files. However, *REEL* will also read any of the chemical input formats, including the residues in the Chemical Components Dictionary (Westbrook *et al.*, 2015 ▸), *via* their three-letter code.

**Figure 1:**
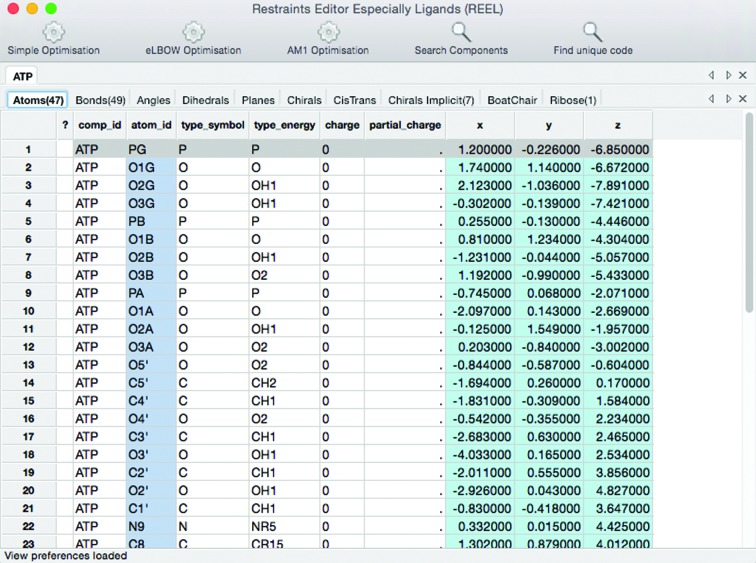
Restraints window of *REEL* displaying the Atoms tab. The color-coding of the cells indicates the interface limitations of the GUI interactions of each cell designed to alert the user to format restrictions and maintain a consistent set of restraints.

*REEL* can be started on the command line as a standalone GUI, but is also available in the main *PHENIX* GUI as a choice in the Ligands menu, as well as being integrated into other program results pages. Upon starting, *REEL* loads the dictionary into a table view with a tab for each restraint type (see Fig. 1 ▸). It also identifies the possible moieties that could be edited. These include *cis*–*trans* isomers, implicit chirals, boat–chair isomers (six-membered saturated rings) and ribose (five-membered saturated rings). Each tab contains a row for each restraint and a cell for each item in the CIF loop that corresponds to it. The rows can be sorted by double-clicking on the column title to toggle the sort order. The estimated standard deviations cells are also colored to indicate warnings and errors (see the Help menu for the legend).

**Figure 2:**
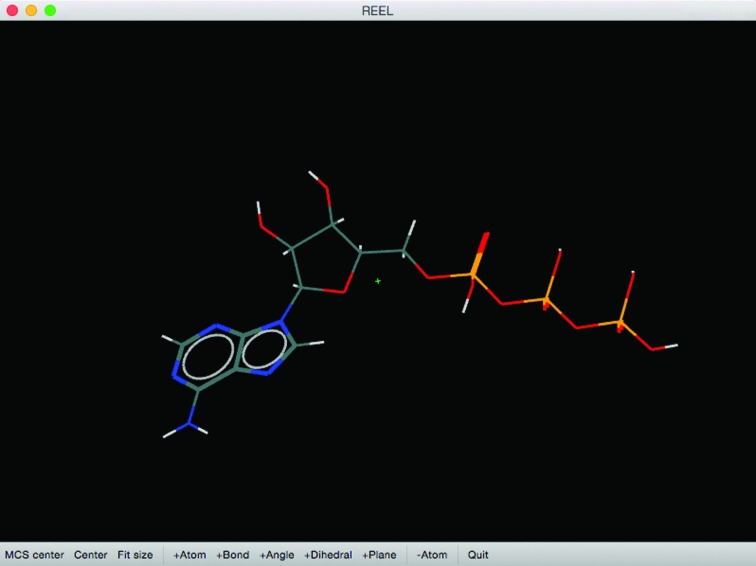
Molecule-view window of *REEL* showing the representation of ATP with bond-order view set to ‘on’. The bottom toolbar can be used for viewing and for molecule and restraints editing.

A second window contains the molecular view (see Fig. 2 ▸). If the restraints contain the Cartesian coordinates, the geometry is displayed in an OpenGL panel that allows rotating, zooming and selections. For restraints files that lack Cartesian coordinates, *REEL* will generate a three-dimensional geometry by selecting the ‘Simple Optimization’ button on the toolbar. An important feature of the molecular view is the reliance of bond specification on the bond restraints in the dictionary input. For formats other than those with bond connectivity, an element-specific distance-based algorithm is used to determine the bonds.

Interaction with the windows makes use of the point-and-click paradigm. Selecting a row in a table view will highlight (in green) the associated atoms in the molecule view, allowing visual feedback on the affected atoms. Color-coding of the cells provides information about the contents, both available actions and validation. The atom names in the atoms tab are colored pale aqua to indicate that editing a cell will propagate the result to all the other instances of that atom name. Aqua indicates that the cell cannot be edited directly. The Cartesian coordinates, if any, are colored in powder blue to indicate that *REEL* only edits the molecular internal coordinates as represented by the restraints. White cells can be changed in either a traditional manner (by typing a new value) or using a selection from a pop-up menu. Changes in these cells only affect that particular restraint and little validation is performed on the resulting restraint.

Selecting an atom or bond in the molecule view will highlight the associated atoms in all table-view restraint tabs. In addition, if the atom is in a plane restraint, the entire plane is highlighted in white. Right clicking^1^ on an atom displays a pop-up menu for some editing procedures such as adding an atom to a plane, changing the atom name or changing the element. The latter two operations are propagated throughout to ensure a consistent set of restraints.

The molecule-window toolbar is situated on the bottom and has some buttons for adjusting the view of the molecule. The molecule-editing buttons are duplicated in the Action menu (see below).

### 3.2. Menu operations   

Pull-down menu items provide access to more global editing options. Some of the options have limited utility but are included for increased flexibility. One goal of *REEL* is to remove the need for text editing the restraint-dictionary file.

#### 3.2.1. File menu   

 
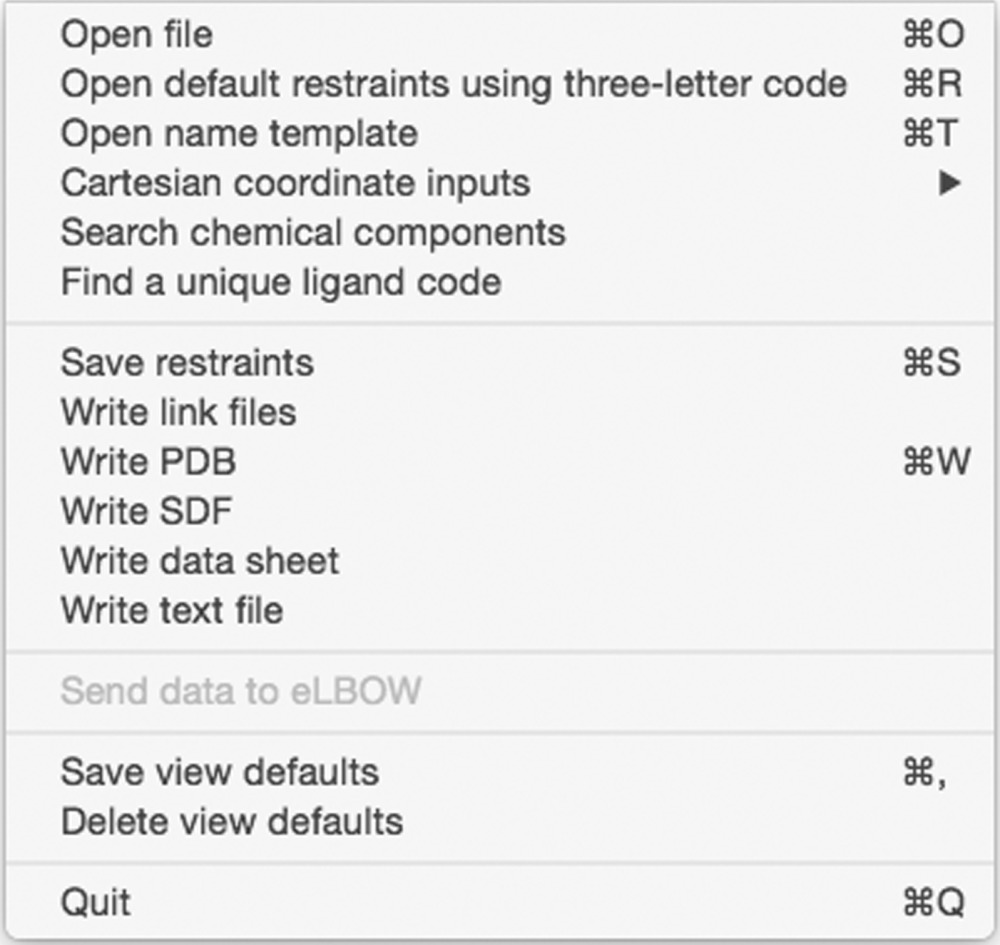


Loading data into *REEL* can be performed *via* the File pull-down menu or *via* options at the command line. Some of the more advanced features that require a command-line start are noted in the corresponding sections below. ‘Open file’ will load any chemical file format that *eLBOW* supports except SMILES. This is most often a dictionary file in CIF format. In the case of a multi-residue PDB-formatted file, the user is presented with a list of residues to select for display. Because *REEL* has direct access to the dictionary-loading machinery in *PHENIX*, the exact restraints for a certain three-letter code can be loaded into *REEL* using the second File menu option for verification of the dictionary or editing of the restraints for use with the current or a different residue. The ‘Open name template’ menu item will use a graph-matching algorithm to rename the atoms in the current molecule with the atoms names in the template file, usually a PDB file. The ‘Cartesian coordinates input’ submenu allows the *xyz* coordinates of the current molecule to be changed with the optional transfer of the corresponding values to the restraints.

Searching the Chemical Components Dictionary or finding a unique, unused ligand code can be performed in this menu or *via* the toolbar buttons. The former is helpful for looking for a desired ligand and opens a window that will show the search results in the molecule window or a text window. The search is a generic text-searching algorithm in the Chemical Components Dictionary including key words and logic such as ‘formula C 1:10’ to search for molecule with one to ten C atoms. Details of the syntax are available *via* the help button on this sub-window. An additional button allows molecules that have a similar Tanimito score (Bajusz *et al.*, 2015 ▸) to be listed using a molecular fingerprint that is a truncated version of the PubChem fingerprint. A detailed description can be accessed at ftp://ftp.ncbi.nlm.nih.gov/pubchem/specifications/pubchem_fingerprints.txt.

Saving the current molecule in a number of formats including restraint-dictionary CIF and PDB model can also be performed using the File menu. The ‘Save restraints’ option writes the restraint dictionary to a CIF-formatted file that is standard for most refinement packages. The final pair of menu items (excluding the ‘Quit’ item) allows the saving and deleting of the current window positions and sizes, and other view options selected in the View menu into the view preferences for *REEL*.

#### 3.2.2. View menu   

 
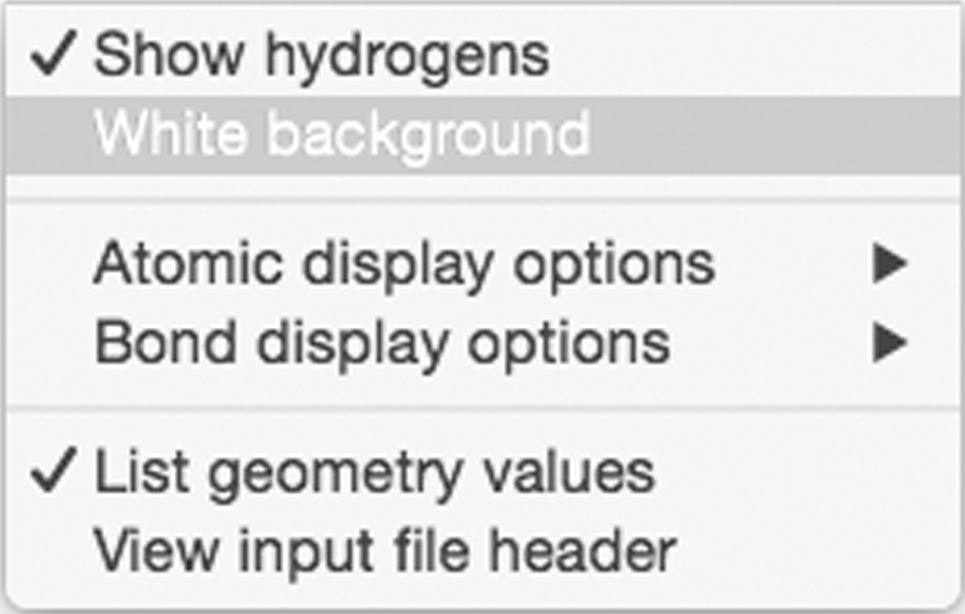


Viewing preferences and options, including showing H atoms, white background, atom and bond attributes such as atom names, and the input file header, can be performed through the View menu. Viewing the input file header can provide additional information, particularly in the case of a Chemical Components CIF, including ligand name, formula and archival dates.

‘List geometry values’ adds a column, or two, to most of the restraints tabs that display the actual geometry value and the difference between the actual value and the ideal value. This is useful option for checking that the restraint is similar to the actual geometry value in some of the options discussed later.

#### 3.2.3. Action menu   

 
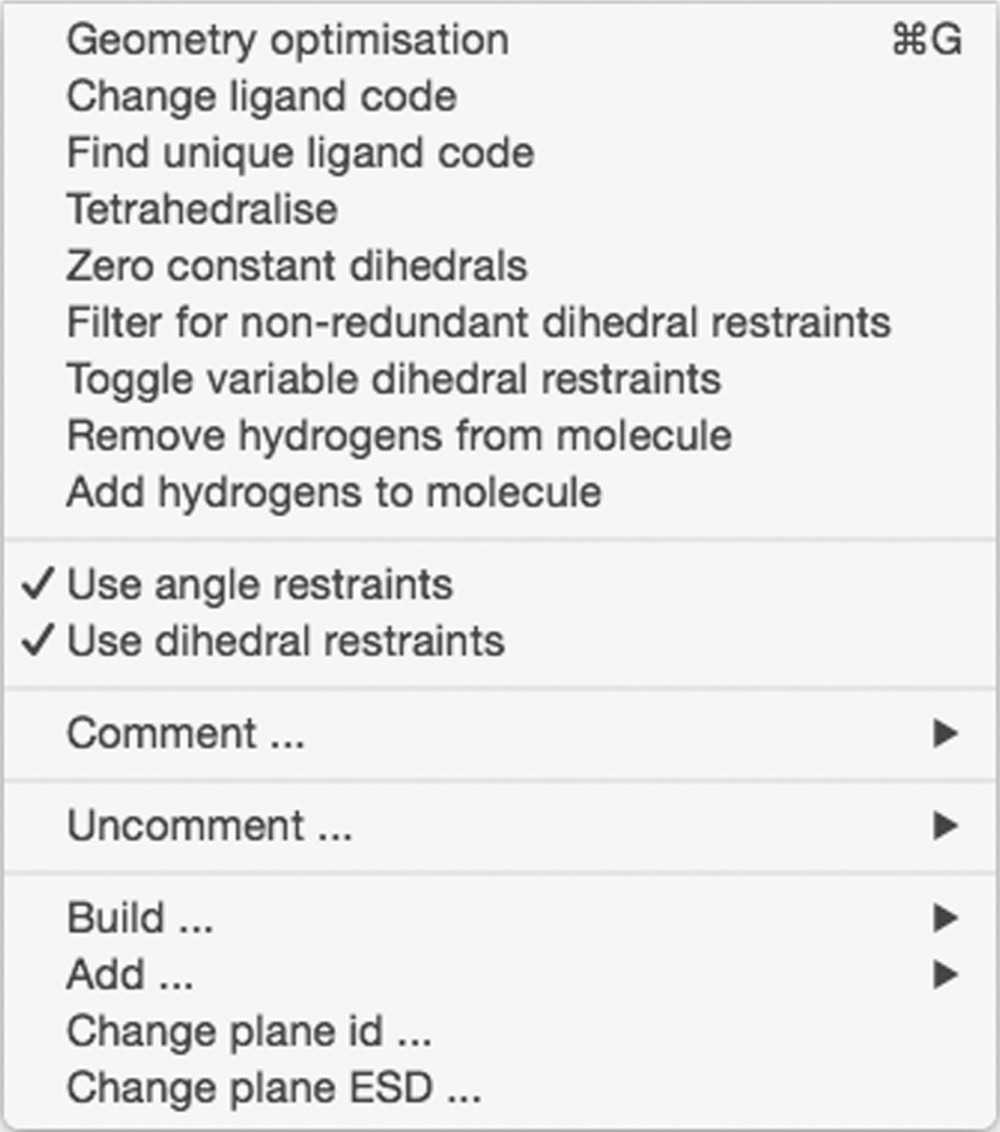


The first item in the Action menu runs a simple geometry optimization on the active molecule using the current, possibly edited, dictionary. This item is useful to provide feedback about what effect the restraint changes will have on the geometry, particularly if they are consistent. Changing the ligand code and finding a unique code can also performed in the Action menu.

By default, *eLBOW* optimizes the geometry with additional options for using the AM1/RM1 semi-empirical quantum-mechanical (QM) method or interfacing with third-party QM packages for higher-level optimizations. Notwithstanding, the ability to idealize the geometry can be performed using the ‘Tetrahedralize’ and ‘Zero constant dihedrals’ menu items.

Other global actions, including and removing H atoms, can be performed in the Action menu. An additional useful action is the deactivation of all angle or dihedral restraints, which is achieved by commenting them and writing the output dictionary file. These deactivated restraints can be removed completely or activated again by the uncomment action in subsequent editing sessions using menu items in the Action menu.

The Add and Build submenus will add atoms and restraints to the molecule. These actions can also be performed using the buttons in the footer of the molecule view. In general, it is more efficient to generate the dictionary from a suitable chemical input in *eLBOW*, but this feature can be helpful for small changes in conjunction with the *eLBOW* menu.

Plane restraints have a different structure to the other restraints and therefore require the last two menu items to change the plane name and estimated standard deviations.

#### 3.2.4. *eLBOW* menu   

 
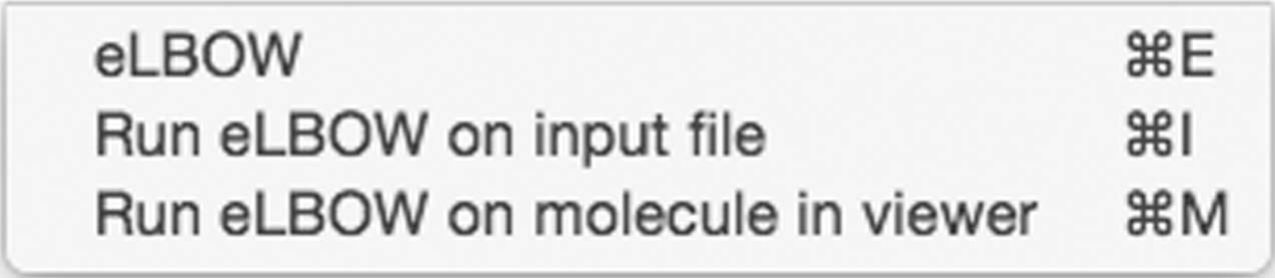


*eLBOW* is the restraint-dictionary generation package available with *PHENIX* and includes a wizard GUI interface in the *PHENIX* GUI. *REEL* also has direct access to the GUI and provides the ability to start an empty *eLBOW* GUI, an *eLBOW* GUI with the input file set to the input given to *REEL* or an *eLBOW* GUI with the input set to the edited current molecule. In all cases, the job is treated as a normal job in the current project of the *PHENIX* GUI.

#### 3.2.5. Sugar menu: overview   

 
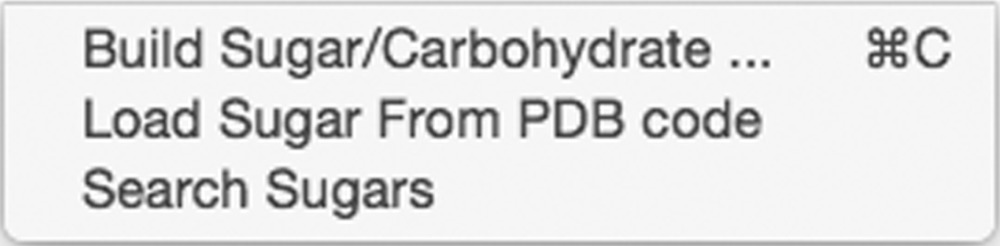


**Figure 3:**
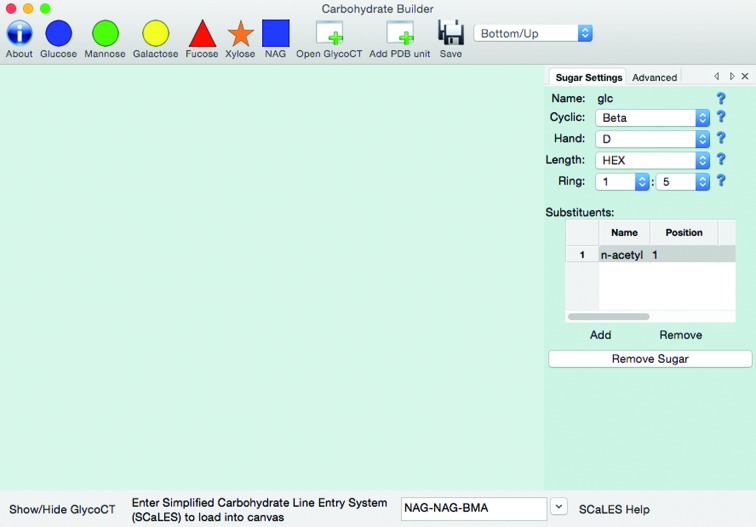
Carbohydrate-building window for creating polysaccharide chains. Input includes a header toolbar with custom buttons and a footer toolbar for SCaLES input. Individual units can be edited using the panel on the right.

Carbohydrates are branched polymers that often attach to the protein covalently. The main feature of the Sugar menu is the ‘Carbohydrate Builder’ shown in Fig. 3 ▸. The base format for describing carbohydrates in *PHENIX* is the GlycoCT format (Herget *et al.*, 2008 ▸). To facilitate the generation of the GlycoCT for the desired complex carbohydrate, a builder with flexible input modes is included in *REEL*.

The builder consists of a header and footer toolbar, a display panel and an editing panel. The toolbars have a number of widgets for flexible carbohydrate-unit addition. There are six buttons for loading common sugar units depicted by their CFG representation (Functional Glycomics Gateway, http://www.functionalglycomics.org/). The units are added to the display panel and linked to the currently highlighted unit using β(1–4) linking. Both carbohydrate units and links can be edited using the editing panel at the right of the builder frame. The attributes of the saccharide that can be changed include cyclic closure, handedness and size. The polysaccharide on the panel can be saved as a GlycoCT file.

A GlycoCT file can be loaded and displayed in the builder for verification and editing as desired. For those familiar with the three-letter codes used by the Protein Data Bank, a button allows the addition of units to the panel using them.

#### 3.2.6. Sugar menu: SCaLES   

To allow more control when building polysaccharides, an inline syntax was developed using similar concepts to the Simplified Molecular-Input Line-Entry System (Weininger, 1988 ▸), known by its acronym SMILES. SMILES was designed to contain all of the information that is required to specify a molecule using the atomic elements as the smallest unit. Some of the important features include a default bond type (single) that can also be specified (‘-’) and branching specified using parentheses.

**Figure 4:**
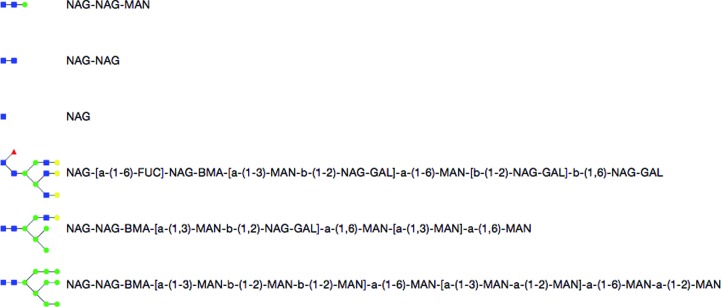
SCaLES examples available in the input-field pop-up menu in the Carbohydrate Builder.

In a similar fashion, the Simplified Carbohydrate Line-Entry System (SCaLES) is designed to contain all of the information to specify a polysaccharide from the PDB codes of each saccharide unit. An unspecified linking is assumed to be β(1–4), which can be specified inline using ‘-b-(1-4)-’. Other links can be specified using the syntax ‘-[a/b]-(n-m)-’ where the ring closure of α and β are specified by a and b, respectively. The numbers n and m are the number of the C atom in the attaching (following) carbohydrate unit and the number of the C atom on the preceding unit, respectively. The examples in Fig. 4 ▸ are available in a pop-up menu of common polysaccharides preloaded into the SCaLES input field at the bottom of the sugar builder.

The SCaLES parser in *PHENIX* has some additional features beyond simply reading the input. The linking of a carbohydrate unit is dependent on the ring closure of the attaching unit. Thus, the linking type must match the ring closure of the attaching unit. One of the most common polysaccharides in proteins is the covalently bound polymer made up of two units of 2-(acetylamino)-2-deoxy-β-d-glucopyranose and one of β-d-mannopyranose. This is commonly erroneously referred to as ‘NAG NAG MAN’. If the SCaLES string ‘NAG-NAG-MAN’ is parsed blindly, the default linking will not match the ring closure of the PDB unit MAN, which is an α-unit. The parser will honour the linking and change the trailing unit to the β-unit, BMA. To specify the polysaccharide without relying on this feature use ‘NAG-NAG-BMA’ or to really link the α-unit use ‘NAG-NAG-a-(1-4)-MAN’.

#### 3.2.7. Sugar menu: carbohydrate search   

This feature allows the searching of several carbohydrate databases including GlycoWorkbench (Ceroni *et al.*, 2008 ▸; Damerell *et al.*, 2012 ▸), GlycomeDB (Ranzinger *et al.*, 2011 ▸ and EUROCarbBD (von der Lieth *et al.*, 2011 ▸) by name in any of the previously defined formats. The carbohydrate can then be added to the builder (if open) for further manipulation and converted to GlycoCT or SCaLES format.

### 3.3. Simple operations   

As already stated, changing an ideal bond value, for instance, can be achieved by editing the appropriate cell in the bond table. The view in the molecule does not automatically update, but a simple geometry optimization can be initiated *via* a button in the toolbar, a pull-down menu (Action → Geometry optimization) or a hot key. In the case of angles and dihedrals, a pop-up menu can be used to change the value to common values such as tetrahedral or 120° for angles and zero or 180° for dihedrals.

Interfacing with chiral restraints is slightly more advanced. Choosing the ‘View → List geometry values’ will display the absolute chiral configuration: *R* or *S*. The absolute configuration is a succinct nomenclature for describing the handedness of a steric center. It is based on the CIP priorities (Cahn *et al.*, 1966 ▸) of the substituents and the direction from high to low priority. The labels are based on the Latin for right (*rectus*) and left (*sinister*).

On the other hand, the chiral restraints are based on the volume described by the three vectors from the central atom to the three non-H-atom substituents. In this schema, the order of the atoms is important. Swapping atoms will change the sign of the volume. The choices (selected by right-clicking on the volume cell) are ‘positiv’, ‘negativ’ or ‘both’, with the latter meaning either ‘positiv’ or ‘negativ’. Selecting a value that requires a change in geometry will update the geometry in the molecule-view window automatically. It is usually the case that the input geometry will determine the chirality if the chirality is not restrained or is set to ‘both’, but there are exceptions. This is owing to the geometry-restraint machinery choosing the nearest minimum as a target.

In addition to the chiral restraints tab, *REEL* has a tab that lists the possible chiral centers based on the geometry of the molecule. This is a simple validation tool for checking that the chiral restraints in the output file are covering the desired chiral centers. If there are additional chiral restraints that are desired, they can be added using the button at the bottom of the molecule-view window.

### 3.4. Complex operations   

Besides tabs for the basic restraints types, bonds, angles, dihedrals, chirals and planes, *REEL* has additional tabs restraining the molecular geometry indirectly. The simplest is the *cis*/*trans* configuration tab. The *E*/*Z* nomenclature is based on the same priorities as the chiral configurations but uses the German word for opposite (*entgegen*) to denote the configuration that has the two highest priority substituents in the *trans* configuration and the German word for same (*zusammen*) for that with the two highest priority substituents in the *cis* conformation. Because there is no restraint for explicitly setting the geometry to a specific isomer and because not all chemical inputs support *cis*/*trans* isomer specification, *REEL* allows this specification. Right clicking on the ‘restrained_to’ cell will allow the selection of either *E*, *Z* or either. The restraint that is performing the work is the dihedral involving the two high-priority substituents across the double bond. If *E* is selected, the dihedral will have an ideal value of 180° and a periodicity of 1. If *Z* is selected, the ideal value is set to zero and the periodicity to 1. The ‘either’ case does not change the ideal value but sets the periodicity to 2, thereby covering both zero and 180°. It should be noted that in the ‘either’ case the input geometry in the model would determine the isomerization of the ligand.

The ‘BoatChair’ tab and the ‘Ribose’ tab both use dihedrals to restrain a flexible ring into the desired conformation. The ‘BoatChair’ tab pertains to the six-membered saturated ring. It can be in a number of conformations, including the so-called ‘Boat’, ‘Chair’ and ‘Twisted’. There are six dihedrals involving the atoms of a six-membered ring. In the ‘Boat’ configuration the signs of the dihedrals alternate and can be denoted ‘+-+-+-’. The first dihedral is defined as the dihedral involving the first atoms listed in the restraints row in the tab. The second moves around the ring by one atom. The pop-up menu in the ‘restrain_to’ cell provides a number of options including the two ‘Chair’ options, six ‘Boat’ options that include two zero dihedrals (denoted, for example, as ‘-0+-0+’) and an option to set to any conformation. Selecting one of the first eight will add six dihedral restraints (visible in the ‘dihedrals’ tab), while the last option removes these six dihedrals. The geometry is automatically updated.

The indirect nature of the ‘BoatChair’ mechanism is repeated for the ‘Ribose’ tab with the appropriate changes in options in the ‘restrain_to’ pop-up menu,

It should be noted that larger or more complex rings could have their confirmations restrained using dihedrals. These dihedrals can be added as required *via* the ‘+dihedral’ button at the bottom of the molecule-view window.

### 3.5. Advanced modes   

After refinement, the geometry of a ligand can differ from the ideal geometry specified by the restraint dictionary used. Large differences indicate that a closer look is required in order to determine the correct geometry. An advanced mode of *REEL* allows some simple validation of the geometry dictionary. At the command line,

phenix.reel ligand.cif refined_geometry.pdb

will load the dictionary and overlay the geometry from the second file. Viewing the actual geometry values and the differences can indicate problem areas for investigation. This operation can also be performed *via* the File pull-down menu options. One can also transfer the geometry values to the ideal values of the restraints.

Another advanced mode of use involves the *PHENIX* .geo file. This file contains all of the restraints, in human-readable format, that were used by the program. This is the definitive list of restraints used in the refinement (reciprocal and real space) and geometry minimization. *REEL* can be used to view the restraints used in a refinement by supplying both the model file and the .geo file.

phenix.reel model.pdb model.geo

will load the model, request the user select the residue of interest and display the residue with only those bonds containing one atom of the selected residue. Selecting a covalently bound ligand will determine if it is indeed bound based on the contents of the .geo file and not on any bond-length cutoff mechanism.

## 4. Discussion   

### 4.1. Implementation concept   

As stated earlier, this restraints editor does not allow direct control of the Cartesian coordinates, but is designed to allow direct and indirect access to the restraint types currently used in macromolecular refinement. Indirect access allows the user to restrain chemical moieties such as saturated rings using the available restraint type (torsion) *via* a quick and easy interface. Interaction with the restraints allows fine-tuning of an individual restraint value and global changes including changing the residue code or removing all dihedral restraints.

### 4.2. Outlook   

More indirect restraints similar to the boat/chair control could be developed in the future, including carbohydrate-specific paradigms which benefit from the alternative angle list for dihedral compared with the nonphysical periodic restraints or the possibly overly restrictive single-value scheme. Ideally, a restraint that allows any chair confirmation and not a boat confirmation (a concerted ring restraint) would be an improvement.

Currently, the definition of indirect restraints relies on the standard restraint types, but additional direct restraint types could be incorporated into a more chemically accurate framework. Small additions have already been introduced into the GeoStd files. These could be extended in the future to provide more accurate geometries and restraint-dictionary deposition to the Worldwide Protein Data Bank.

## Footnotes

1 Mouse clicks are designed assuming a three-button mouse. OSX uses ctrl-click for the right click. Several OS-specific mouse uses can be found in the Help → Mouse controls pull-down menu.
